# Phonons and Phase Transitions in Finite Nuclei

**DOI:** 10.6028/jres.105.020

**Published:** 2000-02-01

**Authors:** N. V. Zamfir, R. F. Casten

**Affiliations:** Yale University, New Haven, CT 06520, USA; Clark University, Worcester, MA 01610, USA; Yale University, New Haven, CT 06520, USA

**Keywords:** nuclear phase transitions, phase coexistence, phonon states

## Abstract

The nature and evolution of collectivity and coherence in nuclei is one of the most fundamental issues in nuclear structure and its evolution with *N* and *Z.* Despite many experiments, the nature of nuclear vibrational modes in deformed nuclei and the nature of nuclear phase/shape transitions are not at all understood. We discuss new experiments on phonon and multi-phonon states in the rare earth nuclei and on new evidence for phase coexistence in Sm that relates to the possible existence of phase transitional behavior in finite nuclei.

## 1. Introduction

This paper addresses two vital issues in nuclear structure. First, despite decades of study, the nature of elementary vibrational, or phonon, modes in nuclei is not at all clear. The question of whether multi-phonon states, especially in deformed nuclei, exist or whether they are fragmented by effects of the Pauli Principle is very much open and, in fact, has only begun to be successfully addressed in the last few years. Secondly, it is commonly thought that phase coexistence and phase transitional behavior in nuclei are unlikely due to the finite number of nucleons and the still smaller number of valence nucleons. Of course, intruder states representing shape coexistence are known but the more fundamental issue we address is that of true coexistence of phases in a one-fluid system.

In this paper, we will address both issues, presenting new data on multi-phonon states in the light rare earth nuclei and new evidence concerning phase coexistence as disclosed by data in the Sm isotopes.

## 2. Phonon and Multi-Phonon States

**Fig. 1:**
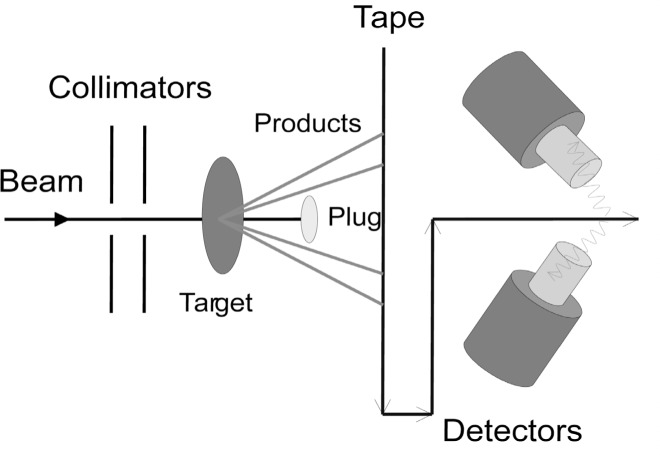
Schematic design of the Yale Moving Tape Collector.

One of the principal research directions in the new nuclear structure program at WNSL at Yale is the study of phonon modes in nuclei, in particular in deformed nuclei, where Pauli fragmentation effects are expected to be larger. The primary instrument for these studies is a Moving Tape Collector (MTC) that collects *β*-activities, produced in-beam following fusion evaporation reactions, and transports them to a low background counting area. The *β*-decay to daughter nuclei gives good access to collective levels, and rather clean spectra. Channel selection to avoid competing activities can often be achieved by the sequencing of tape movements. Data are accumulated in *γ*-multi-scaling and *γ*–*γ* coincidence modes. The first experiments with the MTC used traditional Ge detectors. Current studies exploit the higher efficiency of the clover detectors from YRAST Ball. The MTC, sketched in Fig. 1, uses a novel “plug” design as a high efficiency (≈50 % to 85 %), albeit low beam rejection (≈10^2^) fragment separator: the unreacted primary beam particles are stopped on the plug while most fusion evaporation products by-pass it, and are collected on the tape.

This device is being used for a systematic study of the excited K = 0^+^ excitations in deformed rare earth nuclei. The aim is to investigate if low lying K = 0^+^ excitations are single phonon modes (e.g., *β* vibrations) or 2-phonon double *γ* modes. Evidence (necessary but not sufficient) for the latter is large E2 matrix elements for decay to the *γ* band. Such transitions, though, are difficult to measure because of their low energy (the $E_{\gamma }^{5}$ factor) and careful experiments need to be carried out.

Our first study of these excitation modes did not in fact use the MTC. It focused on ^162^Dy in an off-line experiment in which the (*α*,n) reaction was used to produce ^162^Ho whose *β*-decay half-life is 67 min. Hence it was possible to create individual sources, to perform chemistry on them, and to manually transport them to a counting area. In ^162^Dy, existing experiments [1–3] give completely contradictory results. In one *β*-decay study [1] a strong *γ* ray of 393 keV was observed and placed as a $2_{2}^{+}\left(K=0^{+}\right)\to 4^{+}\left(K=2^{+}\right)$ transition originating in the $2_{2}^{+}$ level at 1453.5 keV. In another study [2] the same *γ* ray was unobserved. In an (n, n*' γ*) study [3] it was also not observed but the authors, based on two (n,*γ*) studies [3,4], considered the *γ*-ray line as a doublet and placed the two *γ* rays depopulating levels proposed at 1574.3 keV and 1575.6 keV.

**Fig. 2:**
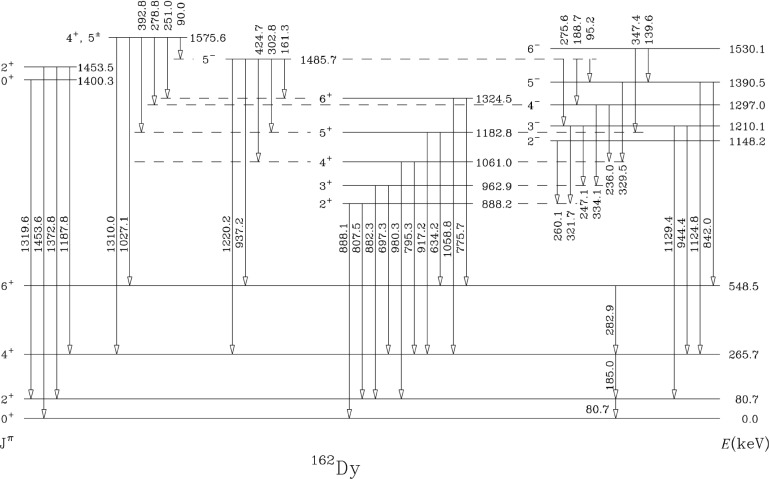
Partial level scheme of ^162^Dy as obtained in this work.

Figure 2 shows a partial level scheme for ^162^Dy, show ing the present results. The experiment performed at WNSL [5] used the (*α*,n) reaction at 19 MeV on a target of ^159^Tb. Data were recorded in singles and *γγ* coincidences. The singles results show clearly that the 392.8 keV line is present as was reported in Ref. [1]. The *γ*–*γ* results, however, show that the placement of Ref. [1] is incorrect and that the 392.8 keV *γ* ray depopulates the level at 1575.6 keV. The spin of this level has not been well established experimentally. From the decay *γ*-ray transitions verified here in the coincidence measurements (transitions to 4^+^, 5^+^, 6^−^, 4^−^, and 5^−^ levels), J^π^ could be reasonably restricted to 4^+^ and 5^±^. If the transition of 278.8 keV to the 4^−^ level at 1297 keV is not correctly placed (the energy is off by almost 0.2 keV), the spin would be limited to 4^+^, 5^±^, 6^−^. This differs from Ref. [3], which proposed 6^−^ from (n,*γ*), but the intensity branching ratios also differ and it is not clear that the same level is being populated. In any case, it is safe to conclude that:

- the 392.8 keV *γ*-ray transition is seen in *β*-decay as observed in Ref. [1].
- it does not deexcite the 2^+^, K = 0^+^ level at 1453.5 keV.
- it does deexcite the level at 1575.6 keV which might be 4^+^, but whose spin is still quite uncertain.

Hence, there is no *positive* evidence for any transition from the $K=0_{2}^{+}$ band to the *γ* band and therefore no evidence, one way or the other, as of yet, for double phonon character. (A 391.5 keV *γ*-ray is assigned in Ref. [3] as a transition from the other level of the doublet, at 1574.3 keV, to the *γ* band based on the (n, *γ*) data they quote as Ref. [4]. However, the intensities for the *γ* transitions from this level given in Ref. [3] are inconsistent with the source (Ref. [4]) that they themselves cite for this data: hence, again no safe evidence exists for a 2-phonon—1-phonon transition.)

A companion study of ^158^Gd [6] at the ILL using the GRID technique [7] to measure the lifetimes of states in the lowest two excited K = 0^+^ bands (and hence the *B* (E2;K = 0^+^ → *γ*) values) has also been carried out. The GRID technique accomplishes two ends. The Doppler broadened line shapes give lifetime information and the ultra-high energy resolution provides nearly certain *γ*-ray placements.

**Fig. 3:**
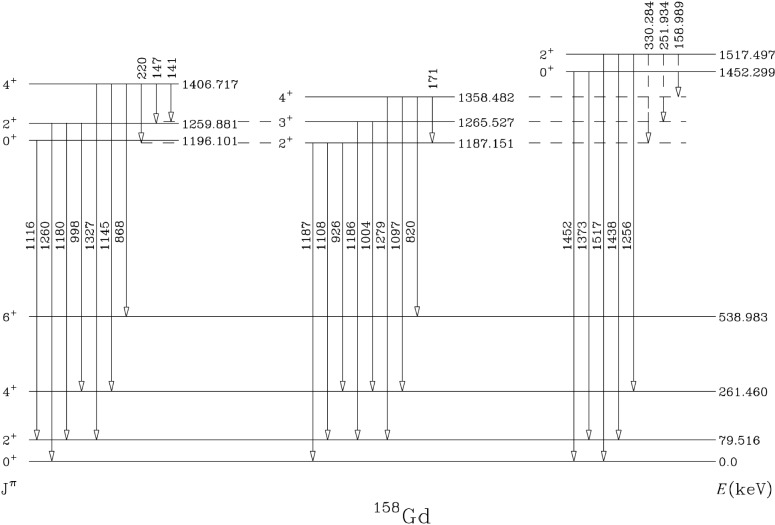
Partial level scheme of ^158^Gd including the present results. Precise *γ*-ray energies are given for a few transitions to aid in following the discussion in the text.

A partial level scheme of ^158^Gd is shown in Fig. 3. There is a strong *B* (E2) value of ≈13 W.u. connecting the $4^{+}\left(K=0_{2}^{+}\right)$ state with the $2_{\gamma }^{+}$ level. Despite the apparently collective nature of this transition, it is actually *not* evidence for 2-phonon character. Indeed, this *B* (E2) value provides an excellent example of a situation where a strong E2 matrix element is only a necessary but not a sufficient condition for assigning *γγ* character to a K = 0^+^ excitation. In this case, states of the same spin in the two bands lie within less than 100 keV of each other and strong bandmixing between them has been shown [8] to fully account for the observed strengths.

Concerning the next K = 0^+^ band with 0^+^ bandhead at 1452 keV, several transitions were placed from the 2^+^ level (1517 keV) of this $K=0_{3}^{+}$ band to the *γ* band in the (n,*γ*) study of Ref. [8], whose authors, however, also speculated on model grounds that the placements might not be correct. We have measured the lifetime of this 2^+^ level and the value obtained, including conservative uncertainties for unknown statistical feeding mechanisms in the radiative capture process, is 0.277 ps < *τ* < 1.478 ps. This lifetime range corresponds to strong *B* (E2) values from the $K=0_{3}^{+}$ band to the *γ* band: for example, 32 W.u. $<B\left(E2;\;\;2^{+}\to 4_{\gamma }^{+}\right)<169$ W.u. These would certainly qualify as collective phonon-like transitions. However, we also made precise energy measurements of a number of *γ* rays occuring in the low lying level scheme of ^158^Gd. From these we deduced the following precise level energies: $E(2_{K=0_{3}^{+}}^{+}=1517.497(3)\mathrm{keV}$, $E(2_{\gamma }^{+})=1187.141\left(3\right)\mathrm{keV}$, $E\left(3_{\gamma }^{+}\right)=1265.520\left(4\right)\mathrm{keV}$, and $E\left(4_{\gamma }^{+}\right)=1358.468(3)\mathrm{keV}$. The level energy differences between the $2_{{K=0}_{3}^{+}}^{+}$ level and the 2^+^,3^+^, and 4^+^ members of the *γ* band differ from the experimental values of Ref. [8] by 62(16), 31(6), and 25(20) eV, respectively. Hence, the placements of the first two transitions cannot be correct. The third transition marginally fits in energy but, without the other two, would very strongly violate the Alaga rules. Again, no safe evidence for *γγ* character of the $K=0_{3}^{+}$ band can be claimed. Note that the energy discrepencies are only a few 10s of eV and that our results therefore require and depend upon the use of the ultra high resolution GAMS4 spectrometer in Grenoble.

Both the ^162^Dy and ^158^Gd examples illustrate an important point, namely that one must be extremely careful with *γ*-ray placements based on singles Ge detector data at excitation energies where the level density is high. To be confident in transition placements, such data must be supplemented either by *γγ* coincidence data (with modern high efficiency *γ*-ray detector arrays) or by ultra-high resolution *γ*-ray spectroscopy (of the type currently possible only at the ILL).

The present results, combined with others on nuclei such as ^166^Er [9,10] and ^168^Er [11] where evidence for 2-phonon K = 0^+^ excitations has indeed been found, and ^164^Dy [11] where the $0_{2}^{+}$ excitation has been shown not to be collective, show that the situation regarding possible K = 0^+^ *γγ* modes in deformed nuclei is complex and that systematic studies will be needed before any broad conclusions can be reached.

## 3. Phase Coexistence and Phase Transitions in Nuclei

**Fig. 4:**
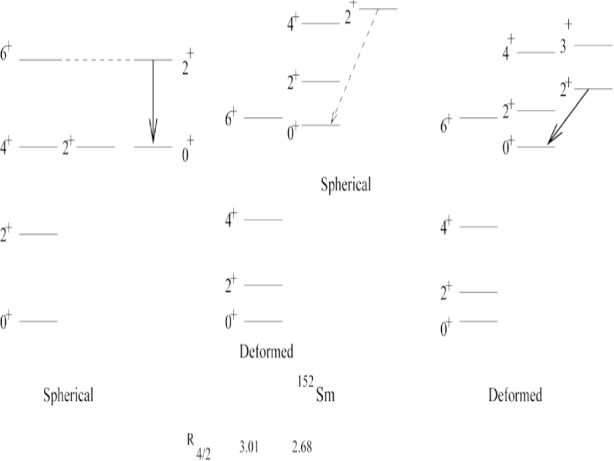
Schematic illustration of the character of the $2_{3}^{+}\to 0_{2}^{+}$ transition for spherical and deformed nuclei (allowed) and in a coexistence picture (forbidden).

**Fig. 5:**
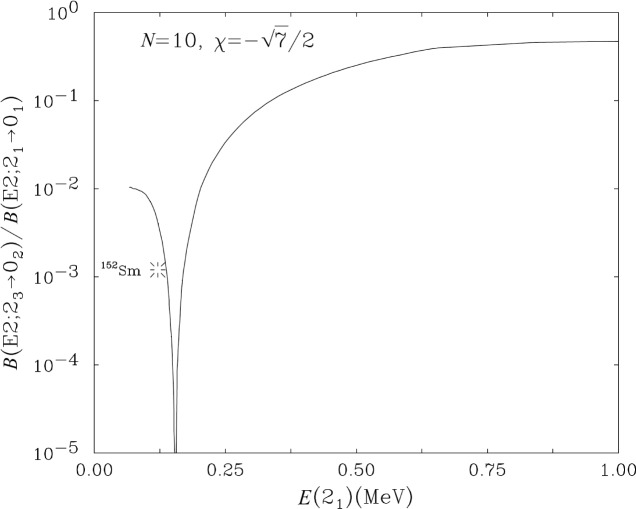
The ratio $B(E2;\;2_{3}^{+}\to 0_{2}^{+})/B(E2;\;2_{1}^{+}\to 0_{1}^{+})$ calculated in the IBA and the experimental upper limit for ^152^Sm.

A recent Köln-Yale experiment [12] on ^152^Sm (*β* decay of ^152^Eu) has shown that the $B\left(E2;\;2_{3}^{+}\to 0_{2}^{+}\right)$ is extremely weak. Specifically, a value of 0.17 W.u. (or less if contaminants are present in the spectra) was obtained, compared with 144 W.u. for the $B\left(E2;2_{1}^{+}\to 0_{1}^{+}\right)$ value. This small value is difficult to understand with traditional paradigms of collective nuclei. As Fig. 4 illustrates, this transition is collective for either a vibrational or a rotational structure. The latter result is perhaps not so familiar but it does in fact result from both the Interacting Boson Model (IBA) [13] and the Geometric Collective Model [14]. In both these models, there is, however, a minuscule region of parameter space where the data are reproduced [12,15]. In this region, the $B\left(E2;\;2_{3}^{+}\to 0_{2}^{+}\right)$ transition actually goes to zero, as region, the $B\left(E2;\;2_{3}^{+}\to 0_{2}^{+}\right)$ transition actually goes to zero, as illustrated for the IBA in Fig. 5. Such a sharp behavior is suggestive of a phase transition. This interpretation is in fact further suggested by the empirical level scheme of ^152^Sm which is illustrated in the middle panel of Fig. 4. Here the yrast levels form a quasi-rotational sequence $\left[E\left(4_{1}^{+}\right)/E\left(2_{1}^{+}\right)=3.01\right]$ while the excited states built above the $0_{2}^{+}$ level form an anharmonic vibrator spectrum $\left[E\left(4_{2}^{+}\right)/E\left(2_{2}^{+}\right)=2.68\right]$. The $2_{3}^{+}\to 0_{2}^{+}$ transition is forbidden in such a scenario since it corresponds to a 2-phonon → 0-phonon transition.

**Fig. 6:**
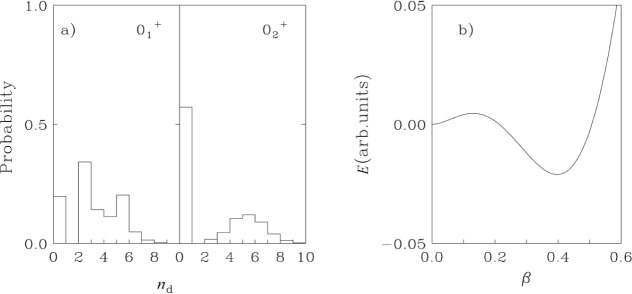
a) Distribution of squared IBA wave function amplitudes for the $0_{1,2}^{+}$ states as a function of the number of d bosons, *n*_d_, in ^152^Sm; b) Energy surface in the intrinsic state formalism, corresponding to ^152^Sm, as a function of the deformation parameter *β*_IBA_.

This interpretation is supported by the IBA calculations. The wave functions of the $0_{1}^{+}$ and $0_{2}^{+}$ levels (expressed in an *n*_d_ basis) are shown in Fig. 6a. The ground state shows a broad distribution typical of a deformed state while the $0_{2}^{+}$ level has almost 60 % probability for *n*_d_ = 0 (ground state of a vibrator). The small amplitudes for higher *n*_d_ states result from mixing of the two phases.

Interestingly, the IBA potential (Fig. 6b) obtained in the intrinsic state formalism for calculations at the minimum in Fig. 5 has two shallow minima, at *β*~0 and for a deformed shape [16]. This kind of phase coexistence is different from that often discussed in the context of intruder orbits (usually with high-j) in the cases of, for example, the Sr-Kr region, the Cd or Hg isotopes, or for superdeformation. In the present case, the coexistence arises in the context of a single Hamiltonian and a single Hilbert space. The sharpness of the phase coexistence region (see Fig. 5) suggests that it is unlikely that any given nucleus falls in the minimum and that this type of coexistence is a rare phenomenon.

Of course, the presence of phase *coexistence* suggests that these nuclei are situated in a phase *transitional* region. This is actually a complex issue since it is commonly thought that true phase transitions cannot occur in a finite nucleus. However, recent work [17,18] has suggested a possible solution to this in which the phase transition is not the property of a single nucleus but rather of a mass region. Such a discussion is beyond the scope of the present paper and we refer the reader to Refs. [17,18].
